# Carvedilol and metoprolol are both able to preserve myocardial function in type 2 diabetes

**DOI:** 10.14814/phy2.14394

**Published:** 2020-03-13

**Authors:** Carol T. Bussey, Aram A. Babakr, Rachael R. Iremonger, Isabelle van Hout, Gerard T. Wilkins, Regis R. Lamberts, Jeffrey R. Erickson

**Affiliations:** ^1^ Department of Physiology—HeartOtago Otago School of Biomedical Sciences University of Otago Dunedin New Zealand; ^2^ Department of Medicine—HeartOtago Dunedin School of Medicine Dunedin Hospital Dunedin New Zealand

**Keywords:** carvedilol, metoprolol, myocardial, Type 2 diabetes, β‐blocker

## Abstract

**Purpose:**

Increasing cohorts of patients present with diabetic cardiomyopathy, and with no targeted options, treatment often rely on generic pharmaceuticals such as β‐blockers. β‐blocker efficacy is heterogenous, with second generation β‐blocker metoprolol selectively inhibiting β_1_‐AR, while third generation β‐blocker carvedilol has α_1_‐AR inhibition, antioxidant, and anti‐apoptotic actions alongside nonselective β‐AR inhibition. These additional properties have led to the hypothesis that carvedilol may improve cardiac contractility in the diabetic heart to a greater extent than metoprolol. The present study aimed to compare the efficacy of metoprolol and carvedilol on myocardial function in animal models and cardiac tissue from patients with type 2 diabetes and preserved ejection fraction.

**Methods:**

Echocardiographic examination of cardiac function and assessment of myocardial function in isolated trabeculae was carried out in patients with and without diabetes undergoing coronary artery bypass grafting (CABG) who were prescribed metoprolol or carvedilol. Equivalent measures were undertaken in Zucker Diabetic Fatty (ZDF) rats following 4 weeks treatment with metoprolol or carvedilol.

**Results:**

Patients receiving carvedilol compared to metoprolol had no difference in cardiac function, and no difference was apparent in myocardial function between β‐blockers. Both β‐blockers similarly improved myocardial function in diabetic ZDF rats treated for 4 weeks, without significantly affecting in vivo cardiac function.

**Conclusions:**

Metoprolol and carvedilol were found to have no effect on cardiac function in type 2 diabetes with preserved ejection fraction, and were similarly effective in preventing myocardial dysfunction in ZDF rats.

## Graphical abstract

In this manuscript, we present data comparing the effects of carvedilol and metoprolol on in vivo and in vitro cardiac function in both diabetic and non‐diabetic human patients, as well as in a rat model of type 2 diabetes (Zucker Diabetic Fatty rats). Our data demonstrate that both carvedilol and metoprolol are able to improve the contractility of cardiac muscle from diabetic humans and rats, as well as preserving cardiac function in both models. Importantly, we saw no extra improvement in individuals or animals that received carvedilol over those who received metoprolol, suggesting that any improvement in health outcomes provided by carvedilol (as seen in the COMET study) are not derived from differences in the contractile function of the cardiac muscle.

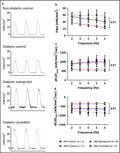

## 1 BACKGROUND

β‐Adrenoceptor (β‐AR) inhibitors are commonly prescribed across a range of cardiac dysfunctions. All β‐blockers inhibit β‐AR activation, maintain calcium homeostasis in myocytes and prevent arrhythmia. However, there is a range of β‐blockers in use, and not all β‐blockers are equal (Haas, Vos, Gilbert, & Krum, 2003; Sica, 2005). Second generation β‐blockers, such as metoprolol, selectively inhibit β_1_‐ARs. Meanwhile, third generation β‐blockers, including carvedilol, nonselectively inhibit β‐ARs with additional inhibition of α_1_‐ARs (Nichols, Gellai, & Ruffolo, 1991), contributing to additional benefits such as improved vascular function. Furthermore, carvedilol is an antioxidant (Yue, McKenna, Lysko, Ruffolo, & Feuerstein, 1992) and inhibits apoptosis (Xu et al., 2014), thereby protecting cardiomyocytes from cellular damage.

These differential effects of β‐blockers has led to the hypothesis that carvedilol is better suited to prevent cardiovascular dysfunction than second generation β‐blockers like metoprolol (Sica, 2005). The Carvedilol Post‐Infarct Survival Control in Left Ventricular Dysfunction (CAPRICORN) randomized controlled trial found that carvedilol reduced cardiovascular and all‐cause mortality after acute myocardial infarction in patients with impaired left ventricular function (Dargie, 2001). This was associated with improved ventricular remodeling and a 3.9% increase in ejection fraction postinfarction (Doughty et al., 2004). The CAPRICORN study further described powerful anti‐arrhythmic effects of carvedilol, as it was able to suppress postinfarction arrhythmias even in patients already treated with an angiotensin converting enzyme (ACE) inhibitor (McMurray et al., 2005). Carvedilol's anti‐arrhythmogenic activity is independent of its role in β‐blockade and is not seen with metoprolol (Zhou et al., 2011). The Carvedilol Or Metoprolol European Trial (COMET) specifically compared the effects of carvedilol and metoprolol tartrate in 3,029 patients with chronic heart failure, and found that carvedilol extends survival compared to metoprolol tartrate (Poole‐Wilson et al., 2003). However, the mechanism underlying improved mortality outcomes in patients receiving carvedilol, compared to those receiving metoprolol, is still unclear.

Further complicating these findings is that, even within the chronic heart failure cohort, diabetes increases the mortality rate by 25% (Haas et al., 2003). Diabetes is linked to a number of cardiac dysfunctions, with apoptosis, oxidative stress, and α‐adrenergic signaling, all contributing to diabetic heart disease (Falcão‐Pires & Leite‐Moreira, 2012; Kamata et al., 2006). Despite the rapidly increasing prevalence of diabetes, there are no specific treatments indicated for diabetic cardiac derangements, and thus β‐blockers are commonly prescribed. Furthermore, scientific investigation has largely focused on heart failure with reduced ejection fraction, but diabetic heart disease often presents with preserved ejection fraction (From, Scott, & Chen, 2010; Palmieri et al., 2001). However, type 2 diabetes confers equivalent cardiac risk regardless of whether ejection fraction is reduced or preserved (MacDonald et al., 2008). These observations suggest that enhanced cardiac risk associated with diabetes may be derived from an underlying perturbation of myocardial function, and previous studies in animal models have demonstrated that contractility is reduced in the diabetic myocardium (Belke, Swanson, & Dillmann, 2004; Daniels et al., 2018; Pereira et al., 2006).

In the present study, we compared the effects of carvedilol and metoprolol on myocardial function in patients and animal models with type 2 diabetes and preserved ejection fraction. We examined cardiac function via echocardiography and myocardial function in isolated trabeculae from patients with and without diabetes prescribed metoprolol or carvedilol, and in Zucker Diabetic Fatty (ZDF) rats following β‐blocker treatment. This approach allowed us to determine whether metoprolol or carvedilol is a more effective β‐blocker to preserve cardiac function at the tissue and whole heart levels, as well as to test the hypothesis that carvedilol confers greater protection against mortality by improving contractility in the myocardium to a greater extent than metoprolol.

## 2 METHODS

### 2.1 Patient characteristics

The local Human Ethics Committee approved the study and all patients provided informed consent. Human epicardial right atrial appendages (RAA) were acquired from coronary artery disease patients with preserved ejection fraction who underwent on‐pump coronary artery bypass graft (CABG) surgery, prior to cardioplegia. Patients requiring emergency CABG, with ongoing myocardial ischemia prior to CABG or those with concomitant cardiac surgical procedures were excluded. All patients had a preoperative transthoracic echocardiogram and functional parameters were determined as previously described (Lamberts et al., 2014). Patients receiving either metoprolol succinate (76.0 ± 6.1 mg day^−1^) or carvedilol (26.7 ± 3.0 mg day^−1^) were grouped into those without (ND) and those with type 2 diabetes (DM), all of whom received clinical diagnosis at least 1 year prior to their surgery.

### 2.2 Human functional force measurements

Myocardial function was assessed in a subset of patients for whom cardiac tissue was available. RAAs were removed under normothermic conditions before cross clamping for cardiopulmonary bypass. Immediately after removal, all specimens were placed in a sealed vial containing a modified Krebs–Henseleit buffer (KHB) (in mM: 118.5 NaCl, 4.5 KCl, 1.4 CaCl_2_, 0.3 NaH_2_PO_4_, 1.0 MgCl_2_6H_2_O, NaHCO_3_ and 11 glucose) with 0.5 mM Ca^2+^ and 6.25 mM 2,3‐butanedione monoxime (BDM) that had been well oxygenated with carbogen (95% O_2_:5% CO_2_). The RAA tissue was immediately returned to the laboratory, such that the dissection of tiny cardiac muscles (trabeculae) commenced 5–10 min after removal. Freshly dissected trabeculae were transferred to an experimental bath and attached between a force transducer and a micromanipulator. Basal and length dependency of force development was measured in the isolated trabeculae, as previously described (Daniels et al., 2018). The muscles were constantly superfused with modified oxygenated KHB, as indicated above but without BDM and at 1.4 mM Ca^2+^, kept at 37°C and continuously stimulated at 60 bpm (1 Hz). To impose similar stretch levels the muscles in both groups were stretched to the length (L_max_) at which isometric developed force (F_dev_) was maximal. Basal F_dev_ was determined following a 1‐hr equilibration.

### 2.3 Animal characteristics

All procedures were approved by the University of Otago Animal Ethics Committee and were conducted in accordance with the New Zealand Animal Welfare Act (1999). Zucker Diabetic Fatty (ZDF) rats have a homozygous missense mutation in the leptin receptor gene (fa/fa) leading to impaired satiety signaling and hyperphagia (Chua et al., 1996; Phillips et al., 1996), and spontaneously develop diabetes from 12 weeks of age due to impaired pancreatic beta‐cell function (Paulsen, Vrang, Larsen, Larsen, & Jelsing, 2010). Obese ZDF rats are a well‐accepted model of type 2 diabetes mellitus, and were compared to their own lean littermates as in‐strain controls. Male rats were bred at the University of Otago from Charles River Laboratories stock (Wilmington, MA, USA), and housed at 20 ± 1°C under a 12‐hr light–dark cycle and provided with food and water ad libitum. All ZDF animals were maintained on Purina 5,008 diet (LabDiet®, St Louis, MO, USA) as recommended by the supplier.

Rats were arbitrarily assigned to receive metoprolol tartrate, carvedilol, or control treatment for 4 weeks from 16 weeks of age. The β‐blockers were of pharmaceutical grade and the dosage was targeted to 100 mg kg day^−1^ metoprolol and 10 mg kg day^−1^ carvedilol, in accordance with clinical dosages and previous studies (Bestetti et al., 1990; Rinaldi et al., 2014). Due to carvedilol's limited solubility, the drugs were delivered orally by crushed into the diet. Concentrations were estimated based on average daily food intake data from previous studies (Bussey & Lamberts, 2017), requiring separate diets for each group, and food intake was monitored to determine delivery.

At the completion of the 4‐week treatment, rats were anesthetized with isoflurane (5% induction, ~2% maintenance; Minrad Inc, Bethlehem, PA, USA) for blood sampling and echocardiography. Blood was sampled from the tail vein for immediate determination of plasma glucose concentrations using a glucometer (Roche, Basel, Switzerland). The upper detection limit of 33.33 mmol L^−1^ glucose may mean that hyperglycemia is underestimated. Blood was centrifuged at 13,000 g for 1 minute and plasma was stored at −20°C for later determination of insulin by ELISA (Millipore, Billerica, MA, USA).

### 2.4 Animal echocardiography

Anesthetized rats underwent a transthoracic echocardiogram, using a Vivid E9 ultrasound system (GE Healthcare, Milwaukee). Standard two‐dimensional echocardiographic left ventricular parameters were obtained from the parasternal short axis, along with pulsed Doppler images of the mitral valve inflow to estimate diastolic function (E/A ratio). Animals were allowed to recover for 1–2 days after echocardiography, prior to terminal procedures.

### 2.5 Rat functional force measurements

Hearts were rapidly extracted from rats anesthetized with pentobarbital (80 mg kg^−1^), and placed in a modified KHB (in mM: 118.5 NaCl, 0.33 NaH_2_PO_4_, 1.0 MgCl_2_6H_2_O, 25 NaHCO_3_ and 11 glucose) with 0.5 CaCl_2_ and 18.5 KCl, and oxygenated with carbogen (95% O_2_:5% CO_2_). The hearts were retrograde perfused via the aorta while cardiac trabeculae were dissected from the right ventricle (dimensions: Length 2.2 ± 0.1 mm, Width 0.3 ± 0.02 mm, Depth 0.1 ± 0.02 mm), mounted on a force transducer, and prepared at L_max_ as described above (Daniels et al., 2018). The muscles were continuously superfused with modified oxygenated KHB as above with 1 mM CaCl_2_ and 4.5 mM KCl at 37°C and stimulated at a basal frequency of 120 bpm (2 Hz). After an equilibration period of 20 min, force–frequency relationships were obtained by measuring steady‐state twitch force conditions at stimulation frequencies of 2, 3, 4, 5, and 6 Hz.

### 2.6 Data and statistical analyses

Functional data were analyzed using Lab Chart 7.0 (AD Instruments, Dunedin NZ). Force values were normalized to the cross‐sectional area of the trabeculae (width × thickness × π). Statistical analyses were conducted with GraphPad Prism (version 7). Differences amongst groups were compared via a two‐way ANOVA followed by a Holm–Sidak post hoc test, except for β‐blockade intake which was assessed by *t*‐test and sex distribution which was assessed via a chi squared test. Force–frequency relationships were assessed by linear regression.

## 3 RESULTS

### 3.1 Patient characteristics

Patients with diabetes undergoing CABG surgery had higher glucose and HbA1c levels compared to patients without diabetes, in line with their diagnosis (Table 1). HbA1c was also significantly higher in patients with diabetes who were prescribed carvedilol compared to those taking metoprolol. Patients were otherwise well‐matched in terms of age, sex, body mass index, and mean arterial blood pressure, with no significant differences observed on the basis of either diabetes diagnosis or prescribed β‐blocker.

**TABLE 1:** Characteristics of patient groups

	Nondiabetes				Diabetes			
	Metoprolol	*n*	Carvedilol	*n*	Metoprolol	*n*	Carvedilol	*n*
Age (years)	65.2 ± 1.7	42	67.9 ± 2.4	20	67.9 ± 2.4	16	64.4 ± 2.9	14
Sex (M:F)	33:9		16:4		11:5		10:4	
BMI (kg m^−12^)	29.3 ± 0.7	42	30.0 ± 1.2	20	29.8 ± 1.2	16	33.4 ± 1.8	14
Glucose (mmol L^−1^)	6.1 ± 0.2	37	6.3 ± 0.4	17	9.2 ± 0.8	10	9.4 ± 0.7	10*
HbA1C (mmol mol^−1^)	37.4 ± 0.6	41	36.9 ± 1.2	15	55.1 ± 3.2	16^♦^	66.5 ± 3.9	13^♦^, ^$^
MAP (mm Hg)	87.2 ± 4.1	42	93.3 ± 3.4	19	85.1 ± 8.8	16	90.9 ± 4.7	13

Note

Characteristics of patients undergoing coronary artery bypass graft surgery, grouped by type 2 diabetes diagnosis and prescribed β‐blocker.

Abbreviations: BMI = body mass index, HbA1c = glycated haemoglobin, MAP = mean arterial pressure.

* Significantly different diabetes versus nondiabetes overall ANOVA,

^♦^ Significantly different diabetes versus nondiabetes within treatment,

^$^ Significantly different metoprolol versus carvedilol, *p* < .05, values are means ± SE.

### 3.2 Patient echocardiography

**FIGURE 1:**
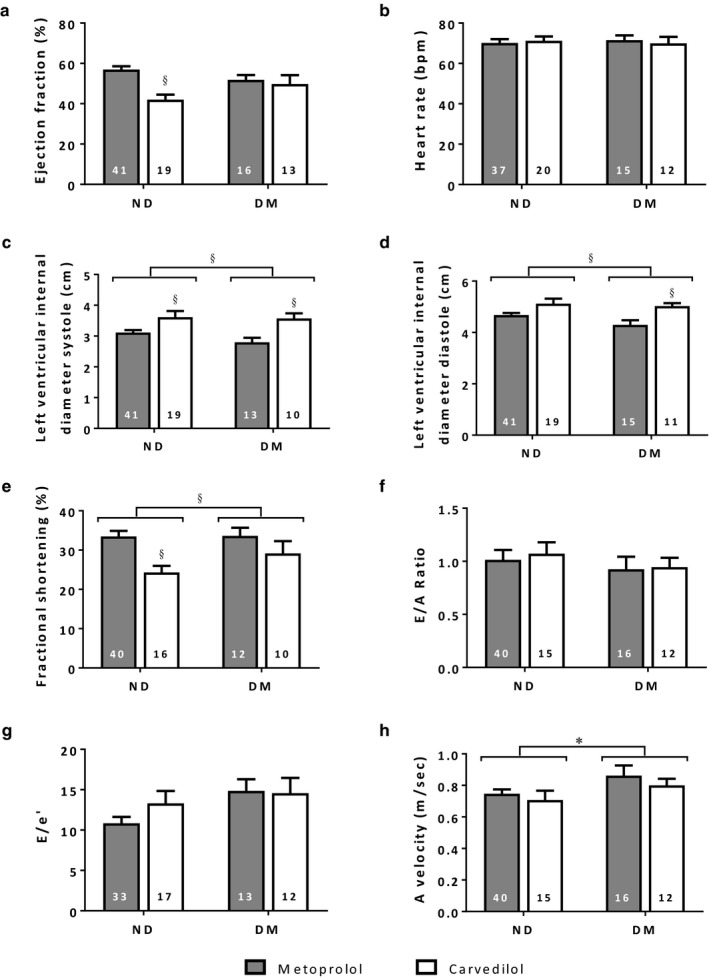
In vivo cardiac function in patients undergoing coronary artery bypass graft surgery, as assessed by echocardiography. *significantly different diabetes versus nondiabetes, § significantly different metoprolol versus carvedilol, *p* < .05, *n* values as indicated within the bars, means ± SE. E/A ratio = the ratio of early (E) to late (A) filling of the left ventricle through the mitral valve, E/e′ = the ratio of early filling velocity (E) and early relaxation velocity (e′), A velocity = velocity of late blood flow from the atrium to the ventricle

Presurgical echocardiographic assessment was clinically indicated for all patients. Analysis of these data indicated mild systolic dysfunction in most groups, with values for ejection fraction at or slightly below 50% (Figure 1a). Most other parameters were within the normal range for the age group, in particular both heart rate (Figure 1b) and E/A ratio (Figure 1f) were not different amongst groups. Compared to nondiabetic patients, those with diabetes had increased A velocity (Figure 1h), and tended toward increased E/e’ (Figure 1g, *p* = .052), suggestive of impaired diastolic function although all values were within the normal clinical range.

Overall, patients prescribed carvedilol exhibited increased left ventricular internal diameter during both systole and diastole (Figure 1c‐d), indicating a potential tendency toward cardiac dilation although values were maintained within the normal range. Fractional shortening was significantly reduced in patients prescribed carvedilol compared to metoprolol (Figure 1e), with the mean value for nondiabetic patients prescribed carvedilol falling below the threshold for mild myocardial contractile impairment (<25%) (Lang et al., 2006). In addition, nondiabetic patients prescribed carvedilol had a significantly reduced ejection fraction compared to nondiabetic patients prescribed metoprolol (Figure 1a).

Taken together, these data suggest that cardiac function is compromised in all patients, unsurprising for a cohort of patients undergoing a CABG procedure, and that patients with diabetes exhibit increased diastolic dysfunction. Patients, both DM and ND, prescribed carvedilol exhibited similar contractile performance to those prescribed metoprolol, with a mild reduction in ejection fraction and fractional shortening in the ND group.

### 3.3 Myocardial function in human tissue

**FIGURE 2:**
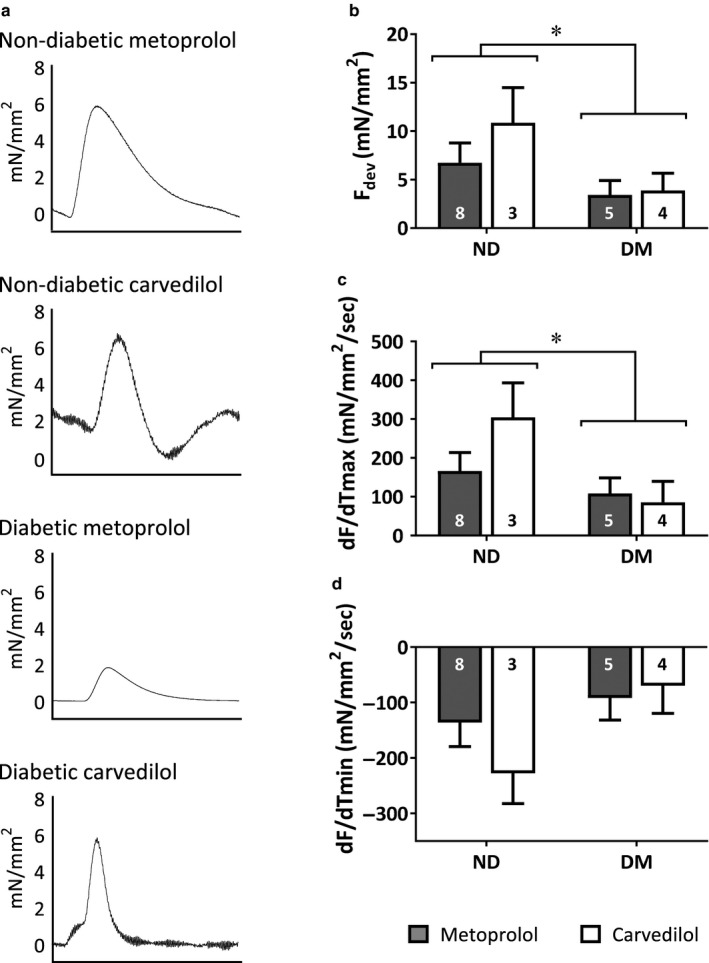
Myocardial function in right atrial appendage trabeculae from patients undergoing coronary artery bypass graft surgery. (a) Representative traces of developed force over 1 second. (b) Force development (F_dev_) and (c) maximal rate of contraction (dF/dT_max_) were reduced in patients with diabetes, with (d) maximal rate of relaxation (dF/dT_min_) also trending toward a decrease (*p* = .066). *significantly different diabetes versus nondiabetes overall ANOVA, *p* < .05, *n* values as indicated within the bars, means ± SE

Myocardial function was assessed in the trabeculae isolated from the right atrial appendage of patients undergoing coronary artery bypass graft surgery (Figure 2a). Trabeculae from patients with diabetes showed significantly reduced F_dev_ and maximal rate of contraction (Figure 2b,c), and a trend toward reduced maximal rate of relaxation (Figure 2d, *p* = .066) compared to patients without diabetes. However, there was no significant difference in any parameter of myocardial function in trabeculae from patients with type 2 diabetes prescribed carvedilol compared to metoprolol. This observation was not consistent with our hypothesis that patients receiving carvedilol would have greater myocardial contractility than patients receiving metoprolol.

### 3.4 Animal characteristics

Our experiments in trabeculae from human patients indicated no differential effects of carvedilol and metoprolol on myocardial and whole heart function in type 2 diabetes. However, these data could not address the ability of the two β‐blockers to preserve cardiac function, as ethical patient care precludes including a group with no intervention. Moreover, all human tissue used in this study was donated by patients undergoing CABG surgery, precluding a healthy control for comparison. Thus, we repeated our experiments in a ZDF rat model of type 2 diabetes.

Basal characteristics of ZDF rats were assessed in 20‐week old animals following 4‐week treatment with metoprolol, carvedilol, or control diet (Table 2). The 20‐week time point was chosen because the ZDF model impaired contractile function but had not severely impaired cardiac function at 20 weeks (Daniels et al., 2018), a good match for our patient cohort. Diabetic rats displayed a characteristic increase in body weight, which was maintained after normalization to tibia length, accompanied by a significant increase in abdominal adiposity as indicated by epididymal fat pad weight. Plasma glucose and insulin levels were also markedly increased in the diabetic ZDF rats, confirming the phenotype. β‐blockade caused a small but significant increase in body weight in nondiabetic animals, with metoprolol and carvedilol having similar effects. However, neither β‐blocker significantly affected any other parameter in nondiabetic rats, or impacted diabetes‐induced changes.

**TABLE 2:** Characteristics and food intake in Zucker Diabetic Fatty (ZDF) rats

	Nondiabetes						Diabetes					
	Control	*n*	Metoprolol	*n*	Carvedilol	*n*	Control	*n*	Metoprolol	*n*	Carvedilol	*n*
Body weight (g)	372.0 ± 5.1	11	390.7 ± 5.3	13^#^	404.8 ± 8.2	12^#^	413.0 ± 7.3	10^♦^	402.1 ± 5.2	16	415.6 ± 6.0	14*
Tibia length (mm)	38.0 ± 1.6	11	37.8 ± 0.5	12	38.4 ± 0.7	10	34.4 ± 1.6	10	35.4 ± 0.8	19	36.4 ± 0.7	12*
Body weight/Tibia length (g mm^−1^)	9.9 ± 0.3	11	10.4 ± 0.2	12	10.4 ± 0.2	10	12.3 ± 0.7	10	11.8 ± 0.4	9	11.4 ± 0.3	12*
Epidydymal fat (g)	2.4 ± 0.2	7	3.1 ± 0.2	8	3.0 ± 0.2	8	7.9 ± 0.3	7	7.8 ± 0.4	7	7.4 ± 0.3	8*
Plasma glucose (mmol L^−1^)	11.5 ± 0.5	11	12.1 ± 1.0	12	12.0 ± 0.6	12	30.9 ± 0.9	10	31.3 ± 0.5	15	31.3 ± 0.6	14*
Plasma insulin (ng ml^−1^)	0.8 ± 0.2	6	0.6 ± 0.2	6	0.8 ± 0.1	6	2.2 ± 0.4	5	1.7 ± 0.2	7	2.1 ± 0.4	6*
Food intake (g day^−1^)	17.9 ± 0.6	11	19.2 ± 0.6	13	19.2 ± 0.5	12	37.6 ± 0.8	10	37.5 ± 1.2	16	39.7 ± 1.8	14*
Metoprolol (mg kg^−1^ day^−1^)			89.3 ± 1.8	13					133.7 ± 4.4	16*		
Carvedilol (mg kg^−1^ day^−1^)					8.7 ± 0.2	12					13.7 ± 0.5	14*

Note

Characteristics of 20‐week‐old Zucker Diabetic Fatty (ZDF) rats and their lean littermates, following 4‐week treatment with approximately 100 mg kg^−1^ day^−1^ metoprolol, 10 mg kg^−1^ day^−1^ carvedilol or control diet. β‐blocker doses are estimated based on daily food intake.

* Significantly different diabetes versus nondiabetes overall ANOVA,

^♦^ Significantly different diabetes versus nondiabetes within treatment,

^#^ Significantly different β‐blocker versus control, *p* < .05, means ± SE.

Food intake was monitored, and calculated per rat per day (Table 2). Rats with diabetes ingested significantly more food per day than their nondiabetic counterparts. This was accounted for within the β‐blocker dosing, such that ND and DM rats were housed separately and prepared chow with different drug concentrations, based on estimated food intake from our previous studies (Bussey & Lamberts, 2017). Actual β‐blocker intake was estimated from the measured food intake for the current study. Calculated β‐blocker doses were significantly lower for ND than DM rats, for both metoprolol and carvedilol. However, neither β‐blocker affected food intake.

### 3.5 In vivo cardiac function

**FIGURE 3:**
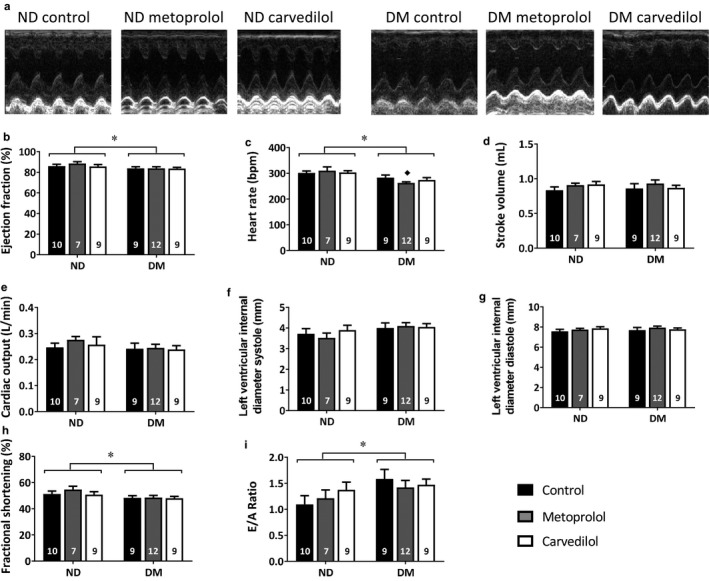
In vivo cardiac function in Zucker Diabetic Fatty (ZDF) rats and their lean littermates, as assessed by echocardiography. Animals were treated with approximately 100 mg kg^−1^ day^−1^ metoprolol, 10 mg kg^−1^ day^−1^ carvedilol or control diet for 4 weeks, after which cardiac function was assessed in 20‐week‐old rats. Representative echocardiograph images, covering a height of 0.5 cm over 1 second, are shown in (a). *significantly different diabetes versus nondiabetes overall ANOVA, ♦significantly different diabetes versus nondiabetes within treatment, *p* < .05, *n* values as indicated within the bars, means ± SE. E/A ratio = the ratio of early (E) to late (A) filling of the left ventricle through the mitral valve

The effects of diabetes and β‐blockade on in vivo cardiac function were assessed by echocardiography (Figure 3a). DM rats displayed small but significant reductions in ejection fraction (Figure 3b) and fractional shortening (Figure 3h) compared to ND, suggestive of impaired contractility although values remained in the normal range. Heart rate was reduced in DM (Figure 3c), in line with our previous studies (Bussey & Lamberts, 2017). However, stroke volume and cardiac output were unchanged (Figure 3d,e). Similarly, left ventricular internal diameter during both systole and diastole was not significantly affected by diabetes (Figure 3f,g). As in the human patients, A velocity was significantly reduced in DM rats (A vel: ND control 52.5 ± 3.7, ND metoprolol 55.7 ± 4.3, ND carvedilol 48.4 ± 3.9, DM control 42.5 ± 5.1, DM metoprolol 43.1 ± 3.2, DM carvedilol 43.8 ± 2.7 cm s^−1^, *p* < .05 ND vs. DM). This contributed to an increased E/A ratio in DM, suggestive of diastolic impairment, although values remained in the normal range (Figure 3i). Furthermore, diabetes may cause mild hypertrophy, suggested by increased ventricular wall thickening in the filling heart (LVPWd: ND control 2.4 ± 0.1, ND metoprolol 2.3 ± 0.1, ND carvedilol 2.3 ± 0.1, DM control 2.6 ± 0.1, DM metoprolol 2.5 ± 0.15, DM carvedilol 2.5 ± 0.1 mm, *p* < .05 ND vs. DM). Taken together, these data demonstrate that cardiac function in the 20‐week ZDF rats was mildly impaired compared to lean littermates, similar to our human cohort.

Chronic β‐blockade had little impact on in vivo cardiac function, with only a further reduction in heart rate in DM animals treated with metoprolol (Figure 3b), and increased interventricular septal thickness at end systole in ND animals treated with metoprolol (IVSs: ND control 3.2 ± 0.1, ND metoprolol 3.7 ± 0.1, ND carvedilol 3.6 ± 0.1, DM control 3.5 ± 0.1, DM metoprolol 3.4 ± 0.1, DM carvedilol 3.3 ± 0.1, *p* < .05 ND metoprolol vs. ND control). Thus, while diabetes led to mild systolic and diastolic impairments, there was no effect of either carvedilol or metoprolol on cardiac function at this early time point.

### 3.6 Ex vivo myocardial function

**FIGURE 4:**
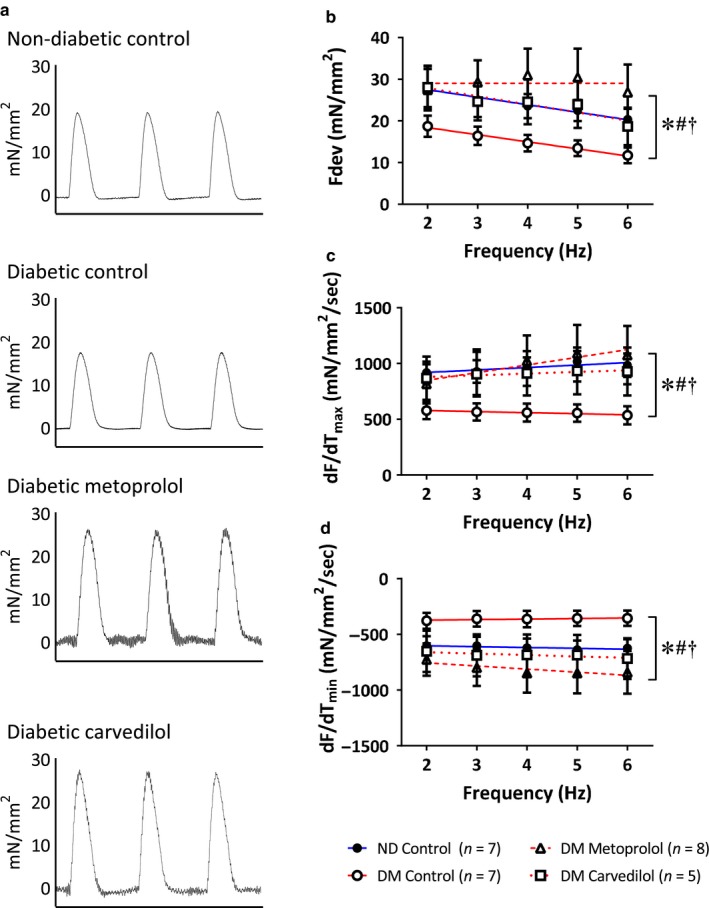
Myocardial function in trabeculae from Zucker Diabetic Fatty (ZDF) rats and their lean control littermates. Animals were treated with approximately 100 mg kg^−1^ day^−1^ metoprolol, 10 mg kg^−1^ day^−1^ carvedilol or control diet for 4 weeks, after which myocardial function was assessed in trabeculae isolated from the right ventricle of 20‐week‐old rats. (a) Representative traces of developed force over 1.5 s. (b) Force development (F_dev_), (c) maximal rate of contraction (dF/dT_max_), and (d) maximal rate of relaxation (dF/dT_min_) were reduced in diabetic animals, and restored with chronic β‐blockade. *significantly different DM control versus ND control overall ANOVA, #significantly different beta‐blocker versus DM control overall ANOVA, † significantly different slope, *p* < .05, values are means ± SE

To assess the myocardial effects of β‐blockade, trabeculae were isolated from the right ventricle of ZDF rat hearts following 4‐week treatment with metoprolol, carvedilol, or control diet (Figure 4a). Type 2 diabetes significantly reduced developed force (F_dev_; Figure 4b), maximal rate of contraction (dF/dT_max_; Figure 4c), and maximal rate of relaxation (dF/dT_min_; Figure 4d) across a range of stimulation frequencies compared to lean, nondiabetic controls. Chronic treatment with either metoprolol or carvedilol restored myocardial function to normal levels for all three contractile parameters but, critically, there was no significant difference between the protective effects of the two β‐blockers. Taken together, our data in both human and rat trabeculae demonstrate that the positive effects of carvedilol on mortality above and beyond metoprolol cannot be attributed to improved contractility of the diabetic myocardium.

**FIGURE 5:**
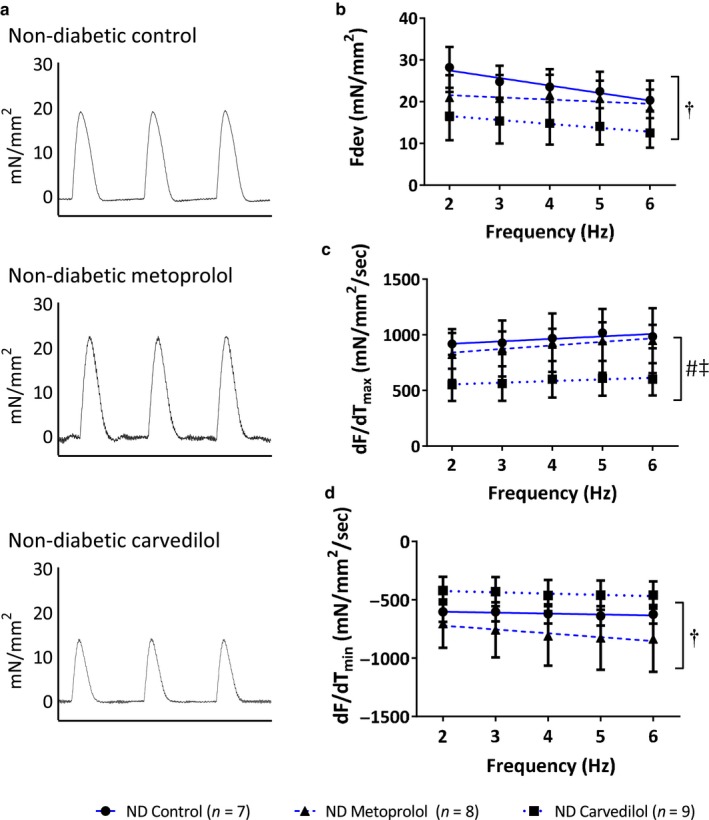
Myocardial function in trabeculae from nondiabetic Zucker Diabetic Fatty (ZDF) rats. Animals were treated with approximately 100 mg kg^−1^ day^−1^ metoprolol, 10 mg kg^−1^ day^−1^ carvedilol or control diet for 4 weeks, after which myocardial function was assessed in trabeculae isolated from the right ventricle of 20‐week‐old rats. (a) Representative traces of developed force over 1.5 s. (b) Force development, (c) maximal rate of contraction (dF/dT_max_), and (d) maximal rate of relaxation (dF/dT_min_) were impaired by chronic β‐blockade in nondiabetic animals. § significantly different metoprolol versus carvedilol overall ANOVA, † significantly different slope, ‡ significantly different intersect, *p* < .05, values are means ± SE

While metoprolol and carvedilol improved myocardial function in diabetes, chronic β‐blockade in ND animals did not increase ex vivo contractility or relaxation (Figure 5a). Indeed, significant differences in the linear regressions suggest that, if anything, chronic β‐blockade in the absence of myocardial impairments was detrimental (Figure 5b‐d). Metoprolol‐treated ND rats had significantly reduced maximal rate of contraction (dF/dT_max_; Figure 5c) compared to those treated with carvedilol.

## 4 DISCUSSION

A lack of suitable, specific treatments hampers outcomes for patients with diabetic cardiomyopathy. It has been suggested that within β‐blocker treatments, carvedilol elicits better outcomes than metoprolol in heart failure, with additional benefits that may extend to type 2 diabetes (Sica, 2005). We hypothesized that these improved outcomes could be explained by enhanced contractile performance in the diabetic myocardium after treatment with carvedilol compared to metoprolol. However, our data lead us to reject this hypothesis, as cardiac trabeculae from both patients undergoing CABG surgery and from ZDF diabetic rats that received carvedilol had no significant difference in contractile function to those treated with metoprolol.

Although we did not identify differential activity of carvedilol and metoprolol, both β‐blockers improved myocardial function compared to untreated type 2 diabetic rats, without an effect on in vivo cardiac function. Our data do therefore suggest that a critical positive effect of β‐blockers in the diabetic heart is the preservation of contractile function, as demonstrated in the controlled rat study. Moreover, it is possible that improved myocardial function may have led to improvements in overall cardiac function had treatment extended beyond the 4‐week period. For this study, we chose to focus on the 20‐week time point, as the ZDF model is severely diabetic with compromised contractility at 20 weeks, but has not yet progressed to severe and potentially irreversible heart failure.

We hypothesized that carvedilol would elicit greater improvements in cardiac function than metoprolol, but where differences in echocardiography measures of CABG patients were found they suggested that patients prescribed carvedilol in fact had poorer cardiac performance. Left ventricular internal diameters during systole and diastole were increased suggestive of dilation, and fractional shortening was decreased across patients both with and without diabetes treated with carvedilol. Patients without diabetes prescribed carvedilol also had reduced ejection fraction compared to those prescribed metoprolol, while carvedilol prescription was associated with increased HbA1c measures in patients with diabetes. However, pathological differences underlying prescription choices cannot be ascertained from the current data, and these differences are likely indicative of differential prescriptions based on clinical criteria rather than a direct effect of carvedilol. In particular, the preferential use of carvedilol in patients with left ventricular systolic dysfunction may reflect both knowledge of the COMET trial and historical restrictions on funding in New Zealand for its use outside the left ventricular systolic dysfunction. All the patients included in the study were undergoing CABG surgery, and were receiving clinically indicated β‐blockers and other medications, which may confound the results. However, heart tissue was not available from healthy controls, as invasive cardiac surgery is a necessary inclusion criterion, and appropriate clinical treatment must be maintained.

Our findings are in contrast with a number of previous studies indicating that carvedilol has greater cardiac benefit than second generation β‐blockers such as metoprolol. Carvedilol decreased all‐cause mortality and improved ventricular remodeling following myocardial infarction compared to placebo in the CAPRICORN Trial (Dargie, 2001; Doughty et al., 2004). Similarly, the Carvedilol Prospective Randomized Cumulative Survival (COPERNICUS) Study showed reduced symptoms, hospitalization and mortality in severe heart failure with carvedilol treatment (Packer et al., 2002). Moreover, the COMET randomized parallel study directly compared carvedilol and metoprolol for treatment of chronic heart failure, also finding a reduction in all‐cause mortality (Poole‐Wilson et al., 2003). Notably, these trials investigated the role of carvedilol in severe cardiac pathology, focusing on high‐risk, postmyocardial infarction patients and chronic heart failure patients with reduced ejection fraction below 35%, respectively. A greater role of sudden cardiac death and arrhythmia in these severe pathologies may be a key difference from diabetic cardiomyopathy (Remme et al., 2007). Furthermore, the COMET trial specifically excluded patients with unstable insulin‐dependent diabetes. In addition, the COMET trial has been criticized both for the formulation of metoprolol (metoprolol tartrate, in contrast to metoprolol succinate used in the current study participants, and tested in MERIT‐HF (Hjalmarson et al., 2000)) and the metoprolol dose, which was approximately half the equivalent carvedilol dose. In the setting of chronic heart failure, β‐blockers are beneficial for patients with diabetes although to a lesser extent than in those with chronic heart failure without diabetes (Haas et al., 2003). In contrast, it has been reported that carvedilol failed to show any beneficial impact on survival or hospitalizations in heart failure with preserved ejection fraction, although that study may lack necessary statistical power (Yamamoto, Origasa, & Hori, 2013). It has been previously suggested that β‐blocker prescription practices should be informed by heart failure stages (Klapholz, 2009). Thus, while we show that carvedilol does not elicit a difference in cardiac function compared to metoprolol in diabetes with preserved ejection fraction, carvedilol may indeed be of greater benefit in more severe pathologies.

While we observed no improvements in cardiac function with carvedilol compared to metoprolol, this does not exclude the possibility that the additional effects of carvedilol may be beneficial in the wider pathophysiology of diabetes. β‐blockers were thought to be contraindicated in patients with diabetes, largely related to negative metabolic side effects, but many of these are avoided by the selection of a third generation β‐blocker such as carvedilol (Bell, 2003; Klapholz, 2009; Kveiborg, Christiansen, Major‐Petersen, & Torp‐Pedersen, 2006; Sica, 2005). Cardiovascular prognosis in patients with diabetes is keenly associated with metabolic control (Kveiborg et al., 2006). Investigating metabolic improvements was not a primary endpoint in the current study, although the results do not indicate a worsening of body weight or glucose in either patients or rats regardless of the β‐blocker. However, metabolic profile has been addressed by the Glycemic Effect in Diabetes mellitus: Carvedilol‐Metoprolol Comparison in Hypertensives (GEMINI) trial, which compared the effects of metoprolol and carvedilol in patients with hypertension and type 2 diabetes. GEMINI found that patients treated with carvedilol had lower HbA1c and improved insulin sensitivity compared to those treated with metoprolol (Bakris, Fonseca, & Katholi, 2004), along with an improvement in patients own perceptions of diabetes‐related symptoms (McGill et al., 2007). Furthermore, carvedilol decreased triglyceride, total cholesterol, and non‐HDL cholesterol levels (Bell, Bakris, & McGill, 2009) and reduced microalbuminuria (Bakris et al., 2005). Meanwhile, treatment with metoprolol was associated with increased weight gain (Messerli et al., 2007) and increased rate and dose of statin therapy (Bell et al., 2009).

Carvedilol may elicit greater metabolic benefits than second generation β‐blockers such as metoprolol through effects outside its β‐blocking capacity. Carvedilol's antioxidant effects have been linked to lowered glucose levels in a streptozotocin‐treated type 1 diabetic rat model (Diogo et al., 2017). Antioxidant properties, along with inhibition of α‐AR and endothelin‐mediated vasoconstriction, may also improve vascular function (Haas et al., 2003). Zhoa *et al.* described vascular benefits of carvedilol through preservation of endothelial junctions, independent of β‐AR inhibition (Zhao, Yang, You, Cui, & Gao, 2007). Furthermore, specific comparison of metoprolol and carvedilol in patients with type 2 diabetes showed a detrimental effect of metoprolol on insulin‐stimulated endothelial function (Kveiborg et al., 2010). As vasodilation, particularly in skeletal muscle, is crucial to glucose disposal (Keske et al., 2017), carvedilol's ability to maintain or improve vascular function may lead to better glycemic control in patients with diabetes than other inhibitors selective for β1‐AR (Haas et al., 2003; Jacob & Henriksen, 2004). Indeed findings in the COMET trial of a 25% reduction in vascular events (stroke or myocardial infarction), alongside a significant decrease in new‐onset diabetes mellitus with carvedilol compared to metoprolol in chronic heart failure (Poole‐Wilson et al., 2003; Remme et al., 2007; Torp‐Pedersen et al., 2007) may signal a key role of vascular improvements in carvedilol's ability to elicit improved outcomes for patients with diabetes.

While both β‐blockers had equivalent effects to offset cardiomyopathy in diabetic rats, they had detrimental effects in nondiabetic controls, both in terms of increased body weight and decreased myocardial function. This pharmaceutical interference in key physiological functions under normal conditions is predictably problematic, and does not reflect clinical practice but was included for a complete set of control conditions. The results highlight the crucial distinction between diseased and control conditions, and the necessary separation from the effects in diabetes that were the primary focus of this study.

The absence of a benefit of carvedilol over metoprolol in type 2 diabetes described herein may relate to the condition studied as discussed, although a number of other differences limit extrapolation. We dosed rats with 100mg kg day^−1^ metoprolol (Bestetti et al., 1990; Rinaldi et al., 2014) and 10mg kg day^−1^ carvedilol (Xu et al., 2014; Yuan et al., 2004) for 4 weeks, similar to previous studies. Pharmacological parameters of β‐blockers vary substantially, so different dosages are commonly tailored to efficacy (Lemmer, Winkler, Ohm, & Fink, 1985). Our animal doses are more disparate than those used for the GEMINI and COMET trials (Poole‐Wilson et al., 2003; Wright et al., 2007), both of which titrated carvedilol doses up to 25mg twice daily, with metoprolol titrated up to 50mg twice daily in the latter, although these are similar to prescriptions in our patient cohort and final doses may be lower. Carvedilol's insolubility necessitated that drugs were delivered in the food, and thus final doses were estimated. While final doses in the animal study were not consistent between nondiabetic and diabetic rats, differential condition‐dependent effects suggest the groups are not comparable and the effect in diabetes was of primary interest. Previous studies, particularly clinical trials, have looked at β‐blocker effects over a longer duration, such as approximately 5‐year follow up for the COMET trial (Poole‐Wilson et al., 2003). It is possible that the effects of β‐blockade on in vivo cardiac function in type 2 diabetes may have emerged given a longer treatment period. However, benefits have previously been described with lower doses of carvedilol for a similar duration (Diogo et al., 2017). The COMET trial also incorporated approximately 1,500 people per group, enabling greater elucidation of subtle differences. However, such large numbers are not feasible for the measures of myocardial function that were a key outcome of the present study. Despite these variables, β‐blockade was effective at preventing myocardial dysfunction in isolated trabeculae, suggesting that the drug delivery was effective.

Furthermore, we describe similar outcomes in human CABG patients as in the controlled animal study. While we conducted similar comparisons of metoprolol and carvedilol in nondiabetic and diabetic conditions, the study segments otherwise had a number of differences. The animal study was strictly controlled, such that diabetic rats received no other medications and their severe hyperglycemia was left uncontrolled. On the other hand, clinical care required that patients were appropriately treated, preventing the inclusion of a control group that received no β‐blocker. In addition, all patients, both with and without diabetes, were undergoing CABG surgery, and were likely comparatively older with a greater duration of diabetes than the ZDF rats specifically assessed at a time of early cardiac dysfunction. A final critical consideration is the use of human atrial tissue and rat ventricular tissue in this study. Previous work by our group in rat cardiac samples showed no difference in functional properties between atrial and ventricular tissue.

Munasinghe et al., 2016). However, we cannot exclude the possibility that β‐blockers have disparate functional effects when comparing the atria and ventricles, particularly in human patients. Despite these confounding factors, confidence in the results is supported by data from both the patient population and controlled animal study being in agreement, with no indication of differential benefits of metoprolol and carvedilol in diabetes.

## 5 CONCLUSIONS

We found no evidence of a difference in cardiac function in type 2 diabetes with carvedilol compared to metoprolol. Both β‐blockers improved myocardial function in diabetic ZDF rats treated for 4 weeks, without an improvement in in vivo cardiac function. Likewise, isolated trabeculae from CABG patients prescribed metoprolol and carvedilol exhibited similar myocardial contractile properties. Therefore, any potential increased benefit of prescribing carvedilol in type 2 diabetes is not attributable to improved cardiac function, and therefore must be explained by other unknown mechanisms.

## 6 ETHICS APPROVAL AND CONSENT TO PARTICIPATE

All work concerning human tissue and data was approved by the Southern Health and Disability Ethics Committee (LRS/12/01/001/AM13), and all patients provided informed consent. All procedures involving animals were approved by the University of Otago Animal Ethics Committee (AEC99/15) and were conducted in accordance with the New Zealand Animal Welfare Act (1999).

## CONFLICT OF INTEREST

The authors declare that they have no competing interests.
